# Bio-Hybrid Hydrogels Incorporated into a System of Salicylic Acid-pH/Thermosensitive Nanocarriers Intended for Cutaneous Wound-Healing Processes

**DOI:** 10.3390/pharmaceutics14040773

**Published:** 2022-04-01

**Authors:** Katarzyna Bialik-Wąs, Małgorzata Miastkowska, Paulina Sapuła, Klaudia Pluta, Dagmara Malina, Jarosław Chwastowski, Mateusz Barczewski

**Affiliations:** 1 Department of Organic Chemistry and Technology, Faculty of Chemical Engineering and Technology, Cracow University of Technology, 24 Warszawska St., 31155 Cracow, Poland; malgorzata.miastkowska@pk.edu.pl (M.M.); paulina.sapula@doktorant.pk.edu.pl (P.S.); 2 Department of Chemical Technology and Environmental Analytics, Faculty of Chemical Engineering and Technology, Cracow University of Technology, 24 Warszawska St., 31155 Cracow, Poland; klaudia.pluta@pk.edu.pl (K.P.); dagmara.malina@pk.edu.pl (D.M.); jaroslaw.chwastowski@pk.edu.pl (J.C.); 3 Institute of Materials Technology, Faculty of Mechanical Engineering and Management, Poznan University of Technology, 24 Jana Pawła II St., 60965 Poznan, Poland; mateusz.barczewski@put.poznan.pl

**Keywords:** bio-hybrid hydrogels, salicylic acid, pH/thermosensitive nanocarriers, drug release, USP4 method

## Abstract

In this paper, the preparation method of bio-hybrid hydrogels incorporated into a system of salicylic acid-pH/thermosensitive nanocarriers to speed up the wound-healing process was developed. This combination creates a dual drug delivery system, which releases the model hydrophobic active substance—salicylic acid—in a gradual and controlled manner for an extended time. Our research team has determined the various properties of bio-hybrid hydrogels based on their physicochemical (swelling degree, and degradation), structural (FT-IR), morphological (SEM), and mechanical (elongation tests) traits. Moreover, empty pH/thermosensitive nanocarriers and their salicylic acid-containing systems were characterized using the following methods: DLS, TG/DTG, and DSC. Additionally, salicylic acid release profiles directly from thermosensitive nanocarriers were compared to the bio-hybrid matrix. These studies were conducted in PBS (pH = 7.4) for 7 days using the USP4 method. To evaluate the antibacterial properties of the obtained materials, the inhibition of growth of *Staphylococcus aureus*, *Escherichia coli*, *Candida albicans*, and *Aspergillus niger*—as the main microorganisms responsible for human infections—were tested. The obtained results indicated that the pH/thermosensitive nanocarrier–salicylic acid system and bio-hybrid hydrogels are characterized by antibacterial activity against both *S. aureus* and *E. coli*.

## 1. Introduction

Hybrid hydrogels constitute a modern form of delivering active substances or genes in the context of biomedical applications, e.g., in the treatment of long-healing wounds [1,2], osteogenesis [3,4], cancers [5,6], myocardial infarction [7], and Parkinson’s disease [8]. They also can be used as biosensors and contact lenses [9]. Bio-hybrid hydrogel materials are multi-purpose systems enabling their controlled release. These types of carriers can consist of a physically or chemically cross-linked polymer backbone as well as nano/polymer micro- or nanocarriers, organic or inorganic encapsulated with drugs [10].

In the literature, there are more and more studies on hybrid hydrogels incorporating silver nanoparticles [11]; active substances, such as salicylic acid [12], ibuprofen [13], and doxorubicin [14]; as well as antibiotics, such as chloramphenicol [15], nitrofurazone, ampicillin, and clindamycin [16]. However, they do not contain a combination of active substances of natural and synthetic origin in the form of a pH- or thermosensitive nanocarrier-drug system, which are incorporated into the hydrogel matrix. Active substances are conjugated, immobilized, or encapsulated in a polymer matrix in such systems. It may be sensitive to changes in the stimulus present in a given environment.

In general, smart polymers can be divided according to their sensitivity to specific environmental stimuli, such as temperature, pH, light, electric, magnetic, acoustic, or electromagnetic fields. There are also the so-called biosensitive polymers, i.e., those reacting to the presence of specific digestive enzymes, antibodies, change of sugar concentration, or other biochemical reactions [5,10,17,18,19]. Thermosensitive polymers are interesting for medical applications because the induction of a temperature change stimulus occurs in a non-invasive manner. Moreover, spontaneous temperature fluctuations occur in the healthy tissue environment and the inflammatory conditions of the body. An aqueous thermosensitive polymeric solution exhibits reversible sol-gel transitions in a temperature range close to the body. It regulates the introduced drug’s release rate and maintains physicochemical stability and biological activity [5,6,20]. The most commonly used thermosensitive polymers include poly(*N*-isopropylacrylamide), poly(*N*,*N*-diethylacrylamide), poly(*N*-vinylacrylamide), poly(*N*-vinylcaprolactam), phosphazene, and polysaccharide derivatives [20,21,22,23]. Considering the use of pH-sensitive carrier-drug systems, it should be emphasized that many examples of combinations of environmentally sensitive substances with drugs are known in medical practice. Some studies have been compiled in the comprehensive review paper by Rizwan et al. [24], citing numerous examples of environmentally sensitive polymers with potential applications in targeted therapy. In contrast, patent US9248192B2 [25] discloses a novel pH-sensitive carrier and method of preparation, pH-sensitive drug, and pH-sensitive drug composition, each comprising the carrier and method of treating or preventing a disease. The disclosed carrier includes at least one pH-sensitive compound selected from the group consisting of a suitable acid and salt and includes at least one amphipathic substance. Similar research on the controlled release of active ingredients from pH-sensitive materials under conditions simulating the stomach was conducted by Qi et al. [26]. They produced a series of pH-sensitive hydrogels based on salecan for controlled delivery of insulin through a copolymerization reaction between salecan and 2-acrylamido-2-methyl-1-propanesulfonic acid. In vitro drug release experiments have confirmed that insulin released from this smart system can also be adjusted according to the pH of the acceptor fluid.

In the present study, salicylic acid (SA), commonly used as a drug in dermatology and cosmetology with proven topical or systemic effects, was used as the model active substance attached to smart polymers. This drug has properties that are useful for the treatment of skin disorders, as it leads to the reduction of clinical lesions, prolongs the remission of symptoms of chronic diseases with a recurrent course, prevents complications, and leads to the complete or partial restoration of skin continuity [27,28,29,30]. SA is a non-steroidal anti-inflammatory drug with an antiseptic and analgesic effect. It is used in the treatment of skin diseases, such as atopic or seborrheic dermatitis and psoriasis. This activity of SA can be divided into disinfecting (1–10%), keratoplastic (10–20%), and inhibiting the multiplication of epidermal cells (20–50%). In addition, salicylic acid has a positive effect on reducing microbial growth through its antiseptic effect [27,31,32,33,34].

In this article, salicylic acid was successfully introduced into a matrix based on two commonly used polymers in polymer hydrogel dressings—sodium alginate and poly(vinyl alcohol) (PVA). Polymers of natural origin, such as anionic alginates, and from synthetic sources, such as PVA, constitute an interesting combination because the presence of alginates ensures less noticeable pain while changing dressings and an optimal, permanently moist medium in the wound, and PVA possesses desirable properties such as biocompatibility and resistance. Both components are non-toxic and biocompatible; however, only their combination gives the material features that must be distinguished by new generation dressing materials [35,36,37,38]. Moreover, the base matrix has also been enriched with *Aloe vera* extract with proven therapeutic properties described in detail by the authors in previous papers [39,40]. Briefly, the health benefits of aloe metabolites are based on the amount and diversity of biologically active compounds with antibiotic and antimicrobial properties, as well as the presence of specific biostimulants that act synergistically with the bioactive components present in the human body [41,42].

This article is a continuation of the authors’ previous research on developing the composition and desired features of a novel third-generation bio-hybrid dressing system [36,39,40,43]. So far, we have designed and tested hydrogels containing only natural active substances added to the base matrix before the cross-linking process. However, the main aim of the presented research is to show a preparation method of the bio-hybrid hydrogel materials containing active substances of natural origin—*Aloe vera*—and additionally from a synthetic source, such as salicylic acid, which was previously incorporated into pH/thermosensitive nanocarriers. We subsequently obtained a dual drug delivery system, which releases the hydrophobic active substance—salicylic acid—in a gradual and controlled manner for an extended time. The paper describes the method of obtaining bio-hybrid hydrogels and presents the detailed properties of the individual components and the final biohybrid, which have a real chance to become a modern dressing material with a smart drug release system.

## 2. Experimental Part

### 2.1. Materials and Methods

Sodium alginate, poly(ethylene glycol) diacrylate (PEGDA) M_n_ = 575 and 700 g/mol (used as a cross-linking agent), *N*-isopropylacrylamide, *N*,*N′*-methylenebisacrylamide, methyl methacrylate, acrylic acid, and salicylic acid (SA) were purchased from Sigma–Aldrich (Darmstadt, Germany). Poly(vinyl alcohol) (M_n_ = 72,000 g/mol), ammonium persulphate employed as an initiator, glycerine, gum arabic, and all salts required to obtain SBF (Simulated Body Fluids) were acquired from POCH SA (Glivece, Poland). Phosphate-buffered saline (PBS, pH = 7.4) was purchased from Chempur (Piekary Śląskie, Poland), and the ethyl alcohol (96%, *v*/*v*) was from Fisher Scientific (Hampton, NH, USA). *Aloe vera* lyophilisate was purchased from a shop with cosmetics and herbal raw materials, Zrób sobie krem, Prochowice, Poland. In this article, we used the following abbreviations: M—basic matrix; M-T-SA—bio-hybrid hydrogel containing the system of thermosensitive nanocarrier-salicylic acid; M-pH-SA—bio-hybrid hydrogel containing the system of pH-sensitive nanocarrier–salicylic acid.

### 2.2. Synthesis of Empty Thermosensitive Nanocarrier (T)

The thermosensitive polymer nanocarrier was obtained by emulsion polymerization of *N*-isopropylacrylamide and *N*,*N′*-methylenebisacrylamide. Briefly, a 0.5% solution of gum arabic was transferred into a three-necked flask and then placed into a glycerin bath. Subsequently, *N*-isopropylacrylamide and *N*,*N′*-methylenebisacrylamide were added and heated up to 70 °C under an inert gas atmosphere and during constant stirring. Then ammonium persulfate was introduced, and the reaction mixture was heated up to 80 °C for 4 h. After that, the obtained thermosensitive nanocarrier was purified via dialysis with a cellulose membrane (MWCO = 14,000 Da) [44].

### 2.3. Synthesis of Empty pH-Sensitive Nanocarrier (pH)

The pH-sensitive polymer nanocarrier was prepared by transferring 0.5% gum arabic solution into a three-necked flask, which was then placed into a glycerin bath. The molar ratio of acrylic acid to methyl methacrylate was 7:3, relative to the total monomer concentration (0.3 mM). Next, 0.1 mL of PEGDA (M_n_ = 575 g/mol) was added and heated to 70 °C under an inert gas atmosphere and during constant stirring. Subsequently, ammonium persulfate was placed in the flask, and the reaction was carried out for 8 h at 80 °C. The obtained pH-sensitive nanocarrier was purified via dialysis with a cellulose membrane (MWCO = 14,000 Da) [45,46].

### 2.4. Encapsulation of Salicylic Acid into Thermo- or pH-Sensitive Nanocarrier (T-SA; pH-SA)

To increase the therapeutic effects and targeting ability of the used medicament, salicylic acid was also encapsulated on a thermo-sensitive or pH-sensitive carrier. The encapsulation process was carried out by adding 50 mg of salicylic acid dissolved in ethyl alcohol to a purified thermosensitive nanocarrier (T) or pH-sensitive nanocarrier (pH) and placed in a round-bottom flask and stirred at a constant speed of 1200 rpm. After 3 h of stirring at room temperature, the drug-nanocarrier system was freeze-dried for 24 h [47,48].

### 2.5. Preparation of the Bio-Hybrid Hydrogels Incorporated into the System of Salicylic Acid-pH/Thermosensitive Nanocarriers

The synthesis of the bio-hybrid hydrogels was mainly based on the previously described method [39,43]—by conventional chemical cross-linking using a 1% solution of ammonium persulfate as an initiator and poly(ethylene glycol) diacrylate (PEGDA, Mn = 700 g/mol) as a cross-linking agent. Briefly, the pH/thermosensitive nanocarriers–salicylic acid system was dispersed in 2% (*w*/*v*) of an aqueous solution of sodium alginate, using a magnetic stirrer at a constant speed. Next, the 5% polyvinyl alcohol solution was then dispersed as well as the *Aloe vera* extract. After that, a constant amount of poly(ethylene glycol) diacrylate (M_n_ = 700 g/mol) (7.5% *v*/*v*) and glycerin (1.7% (*v*/*v*)) were added and stirred. The mixture was heated to a temperature of 70 ± 2 °C, and then 4.4% (*v*/*v*) of ammonium persulfate was dropped. Finally, all mixtures were poured into Petri dishes and then placed on a heating plate with a temperature of 80 °C for 1.5 h and then conditioned for 24 h in ambient conditions [47,48,49]. A drug-free reference sample was also prepared (M). As a result, a transparent bio-hybrid material was obtained.

### 2.6. Characteristic of Empty Thermo/pH-Sensitive Nanocarrier and the System with SA

#### 2.6.1. Dynamic Light Scattering (DLS)

The Zetasizer Nano ZS from Malvern Instruments (Morvin, UK) was used to determine the average size of the obtained thermo/pH-sensitive nanocarrier and the systems with salicylic acid. Samples were analyzed three times at a temperature of 25 °C.

#### 2.6.2. Encapsulation Efficiency

In order to determine the efficiency of the encapsulation, a standard curve of the stock solution and its respective dilutions were prepared. For the solutions obtained, the maximum absorbance was determined at a wavelength of 295 nm. The freeze-dried samples of thermo/pH-sensitive nanocarriers with SA were dissolved in 2% ethanol in PBS, and then the mixture was centrifuged for 10 min at 13,000 rpm. The obtained water phase was subjected to UV-Vis analysis. The encapsulation efficiency is the ratio of the amount of drug that has been incorporated into the nanocarrier (*C_i_*) to the total amount of drug introduced into the carrier during the encapsulation process (*C_t_*). The encapsulation efficiency was determined using Equation (1) [50]:

(1) $$\%EE=\frac{C_{i}}{C_{t}}\cdot 100\%$$

### 2.7. Characteristic of the Bio-Hybrid Hydrogels Incorporated into the System of Salicylic Acid-pH/Thermosensitive Nanocarriers (M-pH-SA, M-T-SA)

#### 2.7.1. Determination of Swelling Degree

The swelling degree (SR%) was evaluated by 10 × 10 mm bio-hybrid hydrogel immersion in excess distilled water, phosphate-buffered saline (PBS, pH = 7.40), or Simulated Body Fluid (SBF, pH = 7.40), with the composition followed by Kokubo and Takadama [51] at 37 °C. The dried and weighed (*W_d_*) bio-hybrid hydrogel samples were soaked in the immersion fluids. After removing the surface water using filter paper, the swollen samples were taken out and weighed (*Ws*) at specific time intervals (after 1, 3, and 24 h). The water uptake of all the tested bio-hybrid hydrogel samples was determined using Equation (2):

(2) $$SR\%=\frac{W_{s}-W_{d}}{W_{d}}\cdot 100\%$$

#### 2.7.2. Degradation Test

Half a gram of each bio-hybrid hydrogel sample weighted with an accuracy of four significant figures was placed in 50 mL of PBS or SBF. The portions were subsequently placed in an incubator at 37 °C. The pH and conductivity values were determined at specific time points for each portion. The studies were carried out for 35 days at ambient temperature and 37 °C, every three days for the first week and later with a one-week time interval.

#### 2.7.3. Attenuated Total Reflection Fourier-Transform Infrared Spectrophotometry (ATR-FTIR)

To identify the chemical structure of the bio-hybrid hydrogels as well as perform an analysis of samples after incubation, attenuated total reflection Fourier-transform infrared spectrophotometry (ATR-FTIR) analysis was done with a Thermo Scientific Nicolet iS5 FT-IR spectrometer equipped with an iD7 ATR accessory in the range of 4000–400 cm^−1^.

#### 2.7.4. Scanning Electron Microscopy (SEM)

The bio-hybrid hydrogels morphology was observed by means of an SEM (Scanning Electron Microscope) using a Tescan Mira 3 instrument equipped with a FEG Schottky electron emission source at an acceleration voltage of 3.0 kV. The samples were sputter-coated with a thin film of gold for 90 s.

#### 2.7.5. Thermal Test (Thermogravimetric Analysis/Differential Thermogravimetric Analysis (TGA/DTG), Differential Scanning Calorimetry (DSC))

Thermogravimetric analysis (TGA) was used to study the thermal decomposition of the used components and final bio-hybrid hydrogels. The 10 ± 0.2 mg samples were heated in the temperature range of 30–800 °C with a 10 °C/min rate, using a Netzsch TG209 F1 apparatus. The measurements were realized using Al_2_O_3_ crucibles in an inert atmosphere (nitrogen). The first mass derivative of the mass degradation (DTG) curves was calculated in reference to the obtained mass vs. temperature curves. The 5, 10, and 50% mass loss (T_5%_, T_10%_, and T_50%_) and residual mass at 800 °C were determined. The thermal properties of the studied materials were analyzed using the differential scanning calorimetry (DSC) method. Samples of 8 ± 0.2 mg were placed in aluminum crucibles with a pierced lid and heated from −50 °C to 290 °C with a rate of 10 °C/min, held at this temperature for 10 min and then cooled back to room temperature with a cooling rate of 10 °C/min. A Netzsch DSC 204F1 Phoenix apparatus and an inert nitrogen atmosphere were used.

#### 2.7.6. Static Tensile Test

The maximum tensile strength and the elongation degree to break tests were performed on the bio-hybrid hydrogels using an MTS Bionix machine with a tensile loading rate of 0.2 mm/s. All specimens were cut into a specific dumbbell shape (75 mm long, 4 mm at the middle, and 25 mm of measuring segment). A film test was performed in a dry state.

#### 2.7.7. Hardness

The bio-hybrid hydrogel’s hardness was tested according to the PN-ISO 868 standard using a type A Shore durometer (Insize Co., Loganville, GA, USA). The measurements were carried out at an ambient temperature, and the data were recorded 15 s after the pressing probe touched the specimen. Each sample was tested in quintuple, and the data are shown as the mean ± standard deviation.

#### 2.7.8. The Release Profiles of Salicylic Acid from Thermosensitive Nanocarrier and Bio-Hybrid Hydrogels

The release of salicylic acid from the thermosensitive nanocarrier and bio-hybrid hydrogels was conducted using the USP4 method (DZF II Flow-Through System, Erweka GmbH, Langen, Germany) [52,53]. The equipment incorporated seven in-line flow-through diffusion cells. The membrane was placed over support with an orifice of 1.5 cm in diameter (diffusional area, 1.766 cm^2^). The vertical cell was made in glass and was designed to have a volume into the donor compartment of 6.22 mL. The cells were placed in a cell warmer connected with an Erweka heater DH 2000i and the Erweka piston pump HKP 720. The piston pump transports the receptor fluid via seven channels to the flow-through cells and automatically adapts the flow-rate setting. All volumes were measured by gravimetric methods by filling the chambers with Milli-Q water and assuming a density of 1 g/mL. All the determinations were made in triplicate for each cell. The release study of salicylic acid was carried out using a regenerated cellulose membrane Spectra/Por^®^Dialysis Membrane MWCO 6-8000 Carl Roth^®^ Company (Karlsruhe, Germany). The assays were performed in 2% ethanol in PBS (pH = 7.40) at a temperature of 37 °C. A flow rate of receptor fluid of about 4 mL/1 min was selected. The experiment was carried out for 144 h. The released concentration of salicylic acid in the receptor solution was analyzed using UV-Vis spectroscopy (Perkin Elmer Company, Waltham, MA, USA) at 295 nm wavelength.

#### 2.7.9. Microbiology Tests

To evaluate the antibacterial properties of the obtained materials, the inhibition growth of *Staphylococcus aureus*, *Escherichia coli*, *Candida albicans*, and *Aspergillus niger* as the main microorganisms responsible for human infections was tested. Growth of the microorganisms was carried out with the use of specific media (TSA for *S. aureus*, McConkey for *E. coli*, Sabourad for *C. albicans*, and YPD for *A. niger*) further incubated for 37 °C for 24 h. After a specified time, inoculates were subjected to liquid growth media and incubated at 180 rpm for 24 h at 37 °C. In the next steps, inoculates were diluted to acquire the optical density (OD) of 1 at 540 nm wavelength using a UV-Vis spectrophotometer [(Rayleigh UV-1800)]. Next, 10 cm^3^ of the respective liquid media was poured into a sterile Petri dish. After a gelling time, 0.1 cm^3^ of diluted bacterial/yeast/fungi was poured. The samples shaped like circular discs with a diameter of 10 mm were placed on the center of the plate and incubated at 37 °C.

## 3. Results

### 3.1. Characteristic of the Empty Thermo/pH-Sensitive Nanocarrier and Their Systems with SA

#### 3.1.1. DLS Analysis

Empty thermo/pH-sensitive carriers and their systems with salicylic acid were subjected to DLS analysis. The average particle sizes of the tested samples are presented in Table 1.

The average particle size of the pH- and thermosensitive carriers is about 479 nm and 118 nm, respectively. These results are very similar compared with the literature [45,46,54]. As a result of the introduction of salicylic acid into the carriers, the average particles size of the system increases up to 700 and 356 nm for the pH- and thermosensitive carriers, respectively. However, this increase did not disturb the monodispersity, and systems with salicylic acid remained stable and did not contain any agglomerates (Figures S1 and S2). The addition of the active substance to the nanocarrier causes an increase in the average particle size because of the deposition of drug particles inside the carrier. This phenomenon does not adversely affect the quality of the obtained samples, and it was confirmed by the literature data [54].

#### 3.1.2. Encapsulation Efficiency

Encapsulation is a physical process that allows drug particles to be immobilized in polymeric carriers. This parameter allows determining the efficiency of the process. The results obtained for nanocarrier–salicylic acid systems are presented in Table 2.

The encapsulation efficiency for the thermosensitive nanocarrier is 80.64%, while for the pH-sensitive nanocarrier, this value is equal to 78.48%. The obtained parameters do not differ significantly, and in both cases, they reach relatively high values. According to the literature data, the encapsulation efficiency of various active substances using a carrier based on the poly(*N*-isopropylacrylamide) polymer is above 70% [54], while for a carrier based on the poly(acrylic acid-co-methyl methacrylate) copolymer is 40–80% [45].

### 3.2. Characteristic of the Bio-Hybrid Hydrogels Incorporated into the System of Salicylic Acid-pH/Thermosensitive Nanocarriers

#### 3.2.1. Swelling Degree

**Figure 1:**
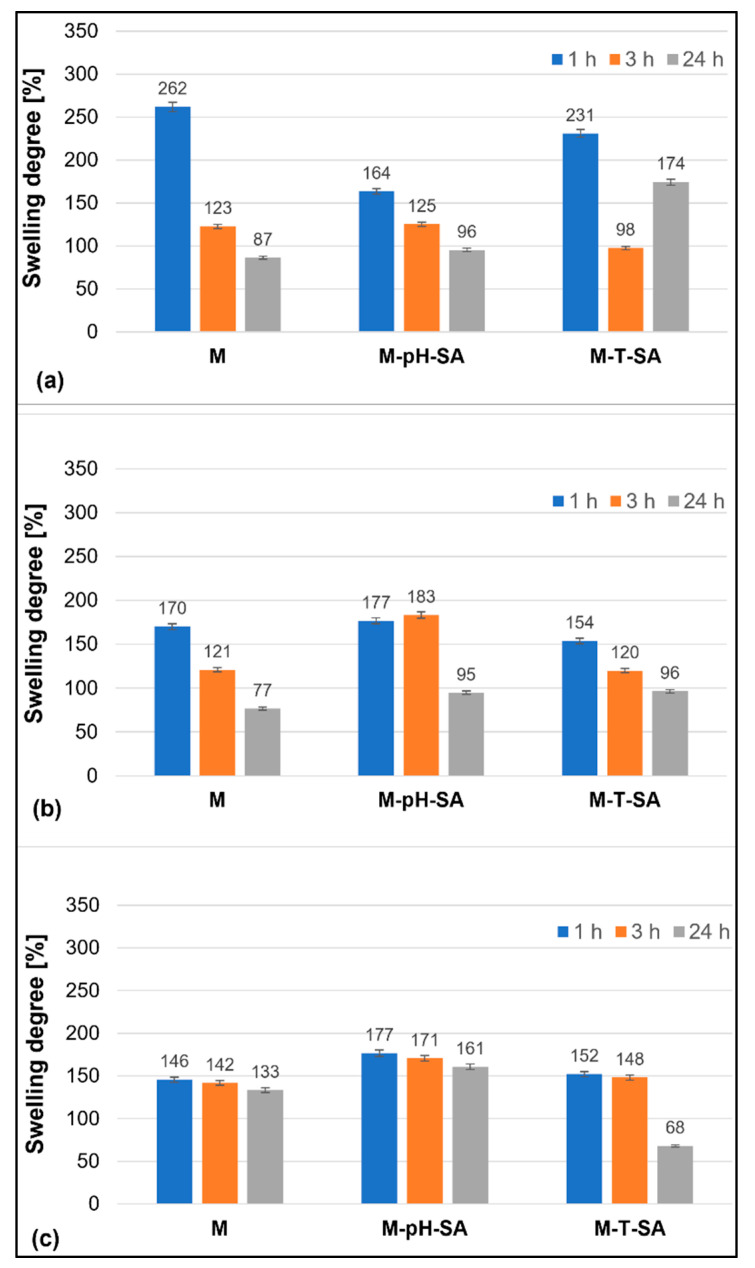
Swelling degree (%) of the basic matrix (M), system with pH-sensitive nanocarrier and salicylic acid (M-pH-SA), and system with thermosensitive nanocarrier and salicylic acid (M-T-SA) at 37 °C of the bio-hybrid hydrogels after tests in (**a**) distilled water; (**b**) PBS, and (**c**) SBF (*n* = 3).

The results of the swelling ability of the tested bio-hybrid hydrogels in different fluids are presented in Figure 1.

The most favorable environment for submerging/storing the tested bio-hybrid hydrogels is the SBF solution, in which only a slight decrease in the SR% parameter is observed with time. The samples containing the pH-sensitive nanocarrier–salicylic acid (M-pH-SA) system show the most stable SR% throughout the study period—the observed decrease in time does not exceed a few percent in comparison with the measurement after 1 h. Similar studies confirm that SBF fluid is a beneficial medium for preliminary bioactivity testing of potential biomaterials [55,56,57]. In the case of the M-T-SA sample, with the system of thermosensitive nanocarrier-salicylic acid, after 24 h of incubation in SBF fluid, a more than 2-fold decrease in swelling capacity and a slight matrix disintegration were observed, which is not beneficial for further applications. In contrast, immersion of materials in distilled water, poorer in ions and slightly acidic, leads to a rapid uptake of water by the sample in the first hour of incubation, after which rapid expulsion of the fluid in the material is observed in every analyzed hydrogel. On the other hand, in phosphate-buffered saline, the most favorable swelling parameters are achieved by M-T-SA bio-hybrid hydrogels, where a gradual decrease in the SR% parameter over time is observed.

#### 3.2.2. Degradation Tests

**Figure 2:**
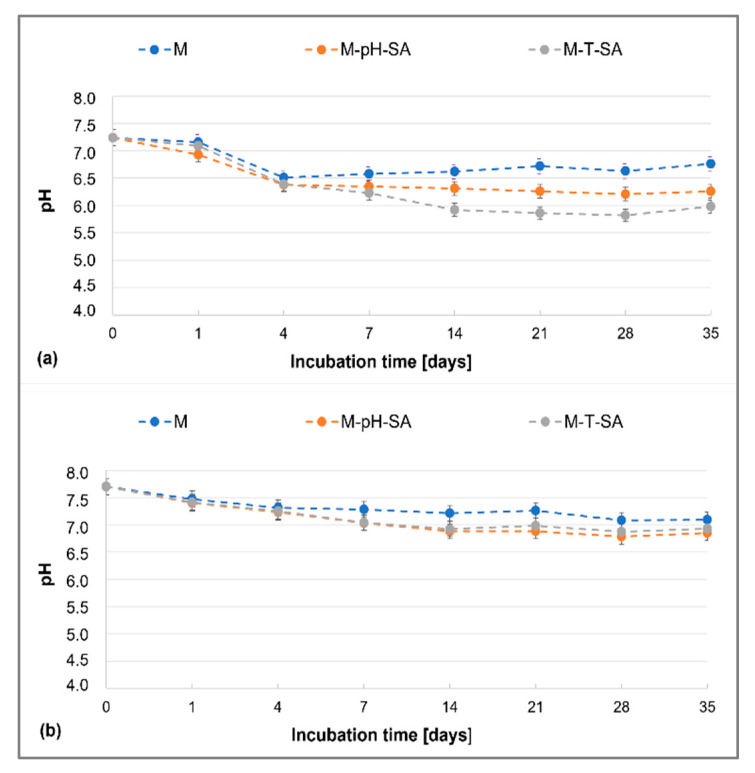
Changes in pH of the basic matrix (M), the system with a pH-sensitive nanocarrier and salicylic acid (M-pH-SA), and system with a thermosensitive nanocarrier and salicylic acid (M-T-SA) at 37 °C during 35-day tests of bio-hybrid hydrogels in (**a**) PBS and (**b**) SBF (*n* = 3).

Analysis of the pH of the fluids in which the obtained bio-hybrid hydrogels and basic matrix were incubated, without modification of the carrier–drug system (Figure 2), shows that the human body-like environment (PBS and SBF), due to its strong buffering properties, maintains a nearly constant pH value throughout the study period, within the neutral pH range. By the fourth day of incubation, the systems gradually reach equilibrium, and both in the case of the pure matrix and the matrix loaded with the smart drug carrier, the observed changes can be neglected by the end of the experiment. The systems are stable over time and do not cause changes in the pH of the medium; however, the SBF medium seems to be the most favorable. By the fourth day of incubation, the systems gradually reach equilibrium, both in the case of the pure matrix and the matrix loaded with the smart drug carrier, and then by the end of the experiment, the observed changes can be neglected—the systems are stable over time and do not cause changes in the pH of the medium, with the SBF liquid being the most favorable medium. It is worth noting that the presence of the carrier–drug system slightly lowers the pH of the fluids, simulating a living organism, which is related to the fact that the salicylic acid was released [58]. On the other hand, the type of carrier used shows no significant effect on the pH of the fluids. The authors observed similar trends in research on a sodium alginate/poly(vinyl alcohol)-based matrix as a basis for studies on the incorporation of the active substance into the matrix presented in this paper [43].

**Figure 3:**
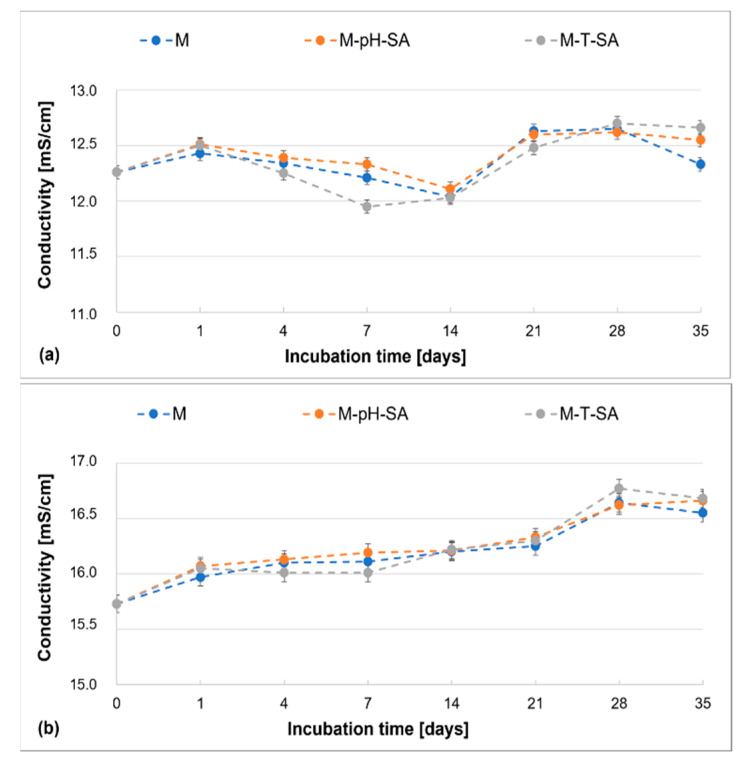
Changes in conductivity of the basic matrix (M), the system with a pH-sensitive nanocarrier and salicylic acid (M-pH-SA), and system with a thermosensitive nanocarrier and salicylic acid (M-T-SA) at 37 °C during 35-day tests in (**a**) PBS and (**b**) SBF (*n* = 3).

The evaluation of the changes in ionic conductivity over time indicates the expected trends, similar to the results obtained by the authors in previously published research [36,39,43]. It was observed that the immersion of the studied matrices, incorporated into the smart nanocarrier–drug system and without the additive, leads to a systematic and gradual increase in the parameters in time in each fluid (Figure 3).

The increase of conductivity in the entire examined period—35 days—is not more than 1 mS/cm, while the increase at the end of the incubation period confirms the beginning of the matrix degradation process and release of its components into the medium. In PBS and SBF fluids, the presence of a pH/thermosensitive carrier–SA system does not influence the described parameter.

#### 3.2.3. FT-IR Analysis

**Figure 4:**
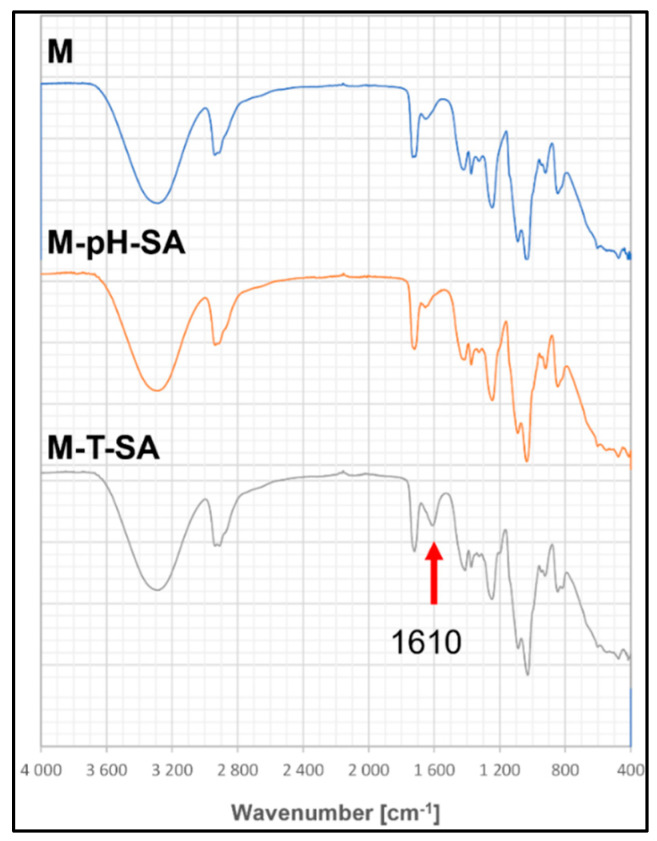
FT-IR spectra of the basic matrix (M), the system with a pH-sensitive nanocarrier and salicylic acid (M-pH-SA), and system with a thermosensitive nanocarrier and salicylic acid (M-T-SA).

The analysis of FT-IR spectra of the base matrix and the bio-hybrid hydrogels containing the pH-sensitive carrier with 50 mg of salicylic acid as well thermo-sensitive carrier, showed slight differences in the intensity of individual absorption bands, which results from the small amounts of the introduced modifier (Figure 4).

All spectra show wide and intense bands in the region of ~3300 cm^−1^, which is associated with the vibration of stretching -OH bonds occurring in the basic components of the matrix—poly (vinyl alcohol), sodium alginate, and *Aloe vera*. Another observed frequency with a medium intensity at the wavenumber of 2940 cm^−1^ can be attributed to the stretching vibration of the C-H groups. The maxima of the bands located at the wavenumber and 1420 cm^−1^ correspond to symmetrical stretching vibrations of the carboxylate anion (COO-) related to the presence of the alginate copolymer. The vibrational bands appearing at 1246 cm^−1^ and 1036 cm^−1^ refer to the presence of the C-O-C group derived from the glycosidic bonds [59]. The band at 1349 cm^−1^ can be attributed to the CH_2_ wagging vibration. The peaks related to the presence of the PEGDA cross-linker are also seen at 1090 and 1035 cm^−1^, related to the C-O-C stretch vibration [41]. The FTIR spectrum of the matrix enriched with the thermo-sensitive carrier–salicylic acid system (M-T-SA) show a change in intensity and a shift in peak from approx. 1653 cm^−1^ to 1610 cm^−1^, which corresponds to the C=C (phenolic) vibration from salicylic acid (S3).

#### 3.2.4. SEM Analysis

**Figure 5:**
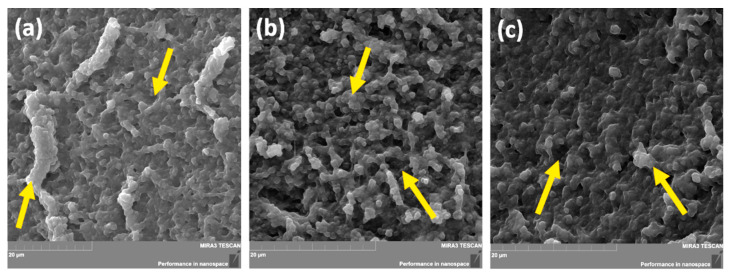
SEM microphotography of the SA/PVA matrix (M) (**a**), with the thermo-sensitive carrier–salicylic acid system M-T-SA (**b**) and pH-sensitive carrier–salicylic acid system M-pH-SA (**c**).

The scanning electron microscopy (SEM) technique has been widely used to investigate a hydrogel’s surface topography and its network structure. As can be seen in Figure 5, the introduction to the polymer system based on sodium alginate and poly(vinyl alcohol) with *Aloe vera* (M), an active substance in the form of salicylic acid on a thermosensitive carrier (M-T-SA), did not cause significant changes in the cross-sectional structure of the analyzed samples. Both micrographs show irregularly shaped structures with comparable size and open pores. This effect may significantly affect the sorption properties, mechanical strength, and release profile of the active substance from the prepared materials. The average pore size of fabricated hydrogels is 0.9 μm to 4.5 μm for the basic matrix (Figure 5a), 1.5 μm–9.3 μm for the thermo-sensitive carrier–SA system hydrogel, and 0.6 μm–4.6 μm for pH-sensitive–SA-loaded hydrogel.

#### 3.2.5. TG/DTG, DSC Analysis

**Figure 6:**
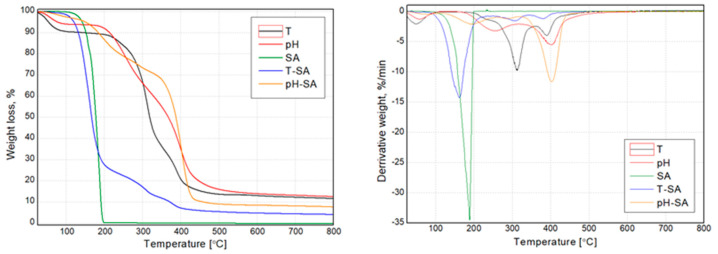
TG and DTG curves of the thermo- and pH-sensitive nanocarriers, salicylic acid, and systems of nanocarriers with SA.

**Figure 7:**
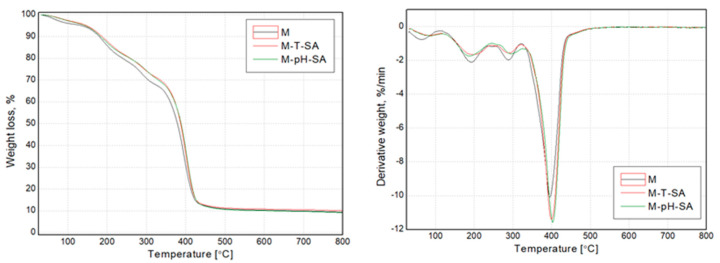
TG and DTG curves of the basic matrix (M) and thermo/pH-sensitive nanocarriers–salicylic acid systems.

The thermogravimetric analysis determined the thermal stability of the functional materials (pH/thermosensitive nanocarriers and salicylic acid) used to modify the hydrogels and final bio-hybrid hydrogel compositions. The results presented as mass (TG) and mass derivative (DTG) vs. temperature curves are presented in Figure 6 and Figure 7. Additionally, selected thermal parameters have been collectively shown in Table 3. Both nanocarriers (pH and T) in unmodified form showed a 3-step thermal degradation process. At the same time, they were characterized by the lowest thermal stability, defined as T_5_. The first degradation step in the temperature range below 100 °C resulted from the hydrophilic character of both materials (T and pH) and evaporation of residual water from their structure. The other two mass loss steps in T and pH are connected with the degradation of the polymeric structure [60,61,62]. SA was characterized by a single-stage sudden process of thermal degradation at the maximum intensity of the process, with reads at the maximum of the DTG curve of 188 °C. Interestingly, a significant effect of the SA supplement on changes in the temperature stability of the carriers was noted. Neither the T-SA nor the pH-SA samples showed the first degradation stage associated with releasing the water residue from the polymeric structure. At the same time, in both cases, a significant reduction in the composition’s temperature stability is seen compared to unmodified materials. In the case of the thermosensitive composition based on cross-linked poly(*N*-isopropylacrylamide) (PNIPAAm), this effect was much more distinct; this material showed a 10% weight loss at the lowest temperature. This may be due to changes in the structure of the polymer due to the presence of SA. Lowering the degradation temperature of the SA-modified thermosensitive nanocarrier may result from lowering the cross-linking density of the PNIPAAm matrix. Increased mobility of macromolecules may cause acceleration of the polymer decomposition processes [61]. The presence of phase separation phenomena resulting from the discrepancy in the miscibility of the T-SA and pH-SA systems cannot be ruled out either. The confirmation of the influence of this effect may be the dominant share of the degradation peak in DTG curve (Figure 6) of T-SA coming from SA, which is not evident in the case of pH- series.

The TG and DTG curves of bio-hybrid hydrogels presented in Figure 7 indicate that salicylic acid-modified nanocarriers increased the thermal stability of the systems compared to the unmodified SA/PVA-based matrix. All the considered systems show a four-stage thermal decomposition, which proves the lack of significant changes in the structure of the lack of inhibiting influence of the modified SA nanocarriers on the formation of the hydrogel structure. It is essential to limit the first step of the decomposition in the range up to 140 °C, which may translate into limiting the effects of water evaporation from the system. This can also be interpreted as an increase in stability during use because the strongly bound water in the hydrogel with limited evaporation will allow its longer-term use. Differences in the subsequent stages of the decomposition process between the modified systems result from differences in the decomposition temperatures of the carriers themselves (Figure 6).

**Figure 8:**
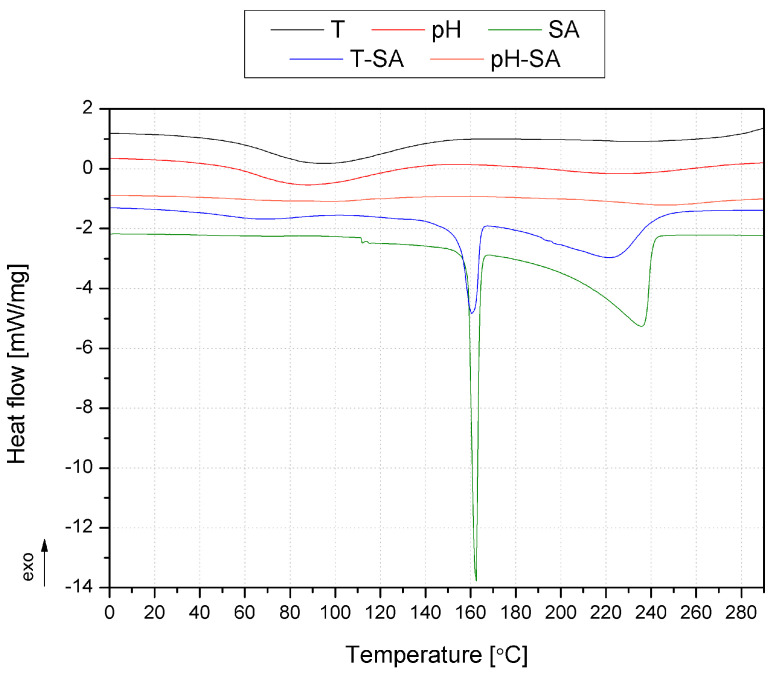
DSC thermograms of the thermo- and pH-sensitive nanocarriers, salicylic acid, and mixtures of nanocarriers with SA.

**Figure 9:**
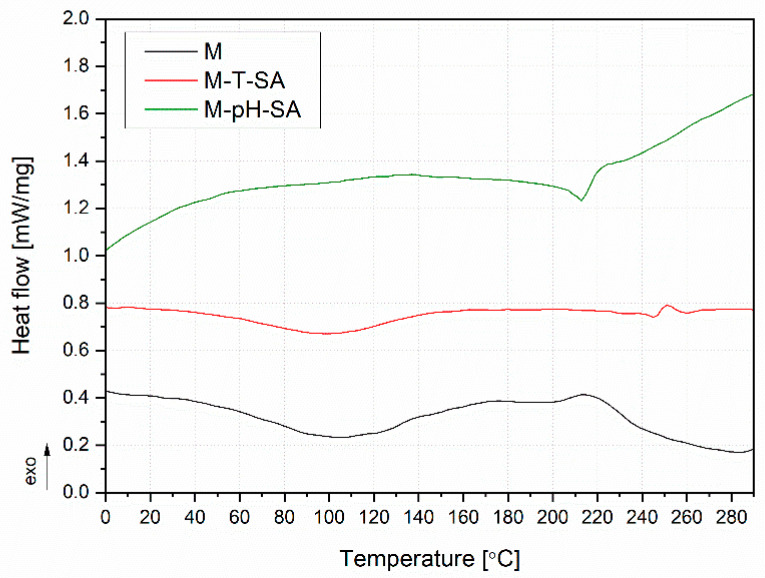
DSC thermograms of the basic matrix (M) and systems with thermo/pH-sensitive nanocarriers and salicylic acid.

The DSC curves are shown in Figure 8 and Figure 9. Figure 8 presents the DSC thermograms of the pH- and thermosensitive nanocarriers, salicylic acid, and systems of nanocarriers with SA. In the case of both nanocarriers, no evident glass transition was noted, which should be recorded for poly(*N*-isopropylacrylamide) (PNIPAAm) at approx. 140 °C [63], on which the T carrier was made, and approx. 76 °C poly(acrylic acid-co-methyl methacrylate) [40]. However, it should be emphasized that, in the case of PNIPAAm, the Tg is strongly related to the molecular mass of the polymer [61], and the determination of Tg in the case of cross-linked polymers is often difficult [60]. Moreover, no exothermic phenomena were noted, indicating a proper cross-linking of polymeric structures. The endothermic peak above 180 °C for pH may be related to the beginning of the degradation process [64]. In the case of the systems (T-SA and pH-SA), two endothermic peaks in the range of 40–140 °C and the other with a peak at 190 and 246 °C were recorded for T-SA and pH-SA, respectively. The first transformation is related to the evaporation of water in the structure of the materials, while the second one relates to the beginning of the phenomenon of the degradation of polymers. The endothermic peak observed in the case of SA and T-SA with a maximum of 156 °C may be attributed to the first-order solid–liquid phase transition of salicylic acid [65,66]. Interestingly, this peak was not observed in the case of the composition with a pH-sensitive nanocarrier. The second endothermic peak at the SA curve in the temperature range of 200–242 °C corresponds to the thermal degradation of the drug described by the TG analysis (Figure 6).

Figure 9 shows the heat flow curves during heating of unmodified (M) and modified hydrogels. The unmodified hydrogels M and M-T-SA showed a broad endothermic peak with a maximum at 98 and 105 °C, respectively, related to water evaporation, as presented in our previous work [39]. In the case of the M-T-SA copy, an additional phenomenon can be noted, which was registered for pure SA (Figure 9), in the same temperature range, of approx. 247 °C. The different course of the M-pH-SA curve may be related to the lower temperature stability of the composition containing the pH-sensitive carrier. No pronounced exothermic peaks could indicate a lack of the hydrogel post-crosslinking process.

#### 3.2.6. Static Tensile Analysis

**Figure 10:**
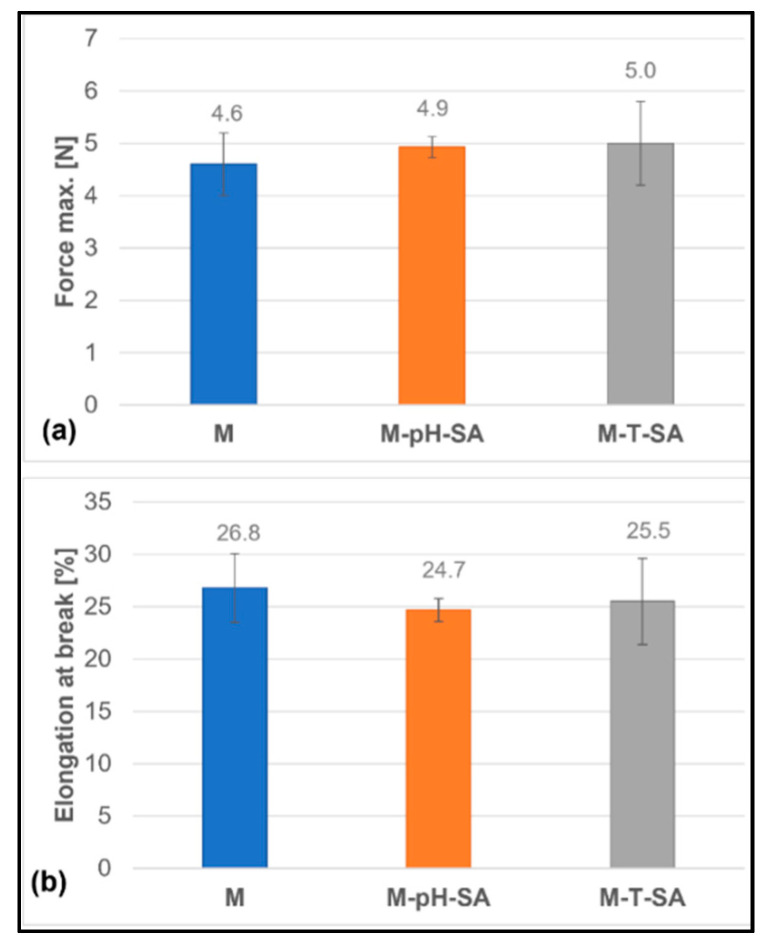
Elongation at break (**b**) and maximum force (**a**) in a static stretching test of bio-hybrid hydrogels (*n* = 5).

The resistance to loading forces of biomedical materials determines their practical application. Hence, providing mechanical stability during use is critical in any material design. It is crucial to conduct strength tests that determine the limit values above which the tested material is weakened or destroyed due to the generated stresses. Mechanical tests have shown that introducing the carrier–drug system into the matrix does not significantly affect the obtained values of the strength parameters (Figure 10). As expected, the maximum force and percentage elongation at the break of bio-hybrid hydrogels were comparable to the corresponding basic matrix due to morphological similarities, including the degree of cross-linking of hydrogels. The determined values of the maximum force are 4.6 ± 0.6 N, 4.9 ± 0.2 N, and 5.0 ± 0.8 N for the basic matrix (M), the drug in a pH-sensitive (M-pH-SA) carrier, and the thermo-sensitive carrier (M-T-SA), respectively. In turn, the values of the maximum elongation at the fracture point of the samples are 26.8 ± 3.3% for M, 24.7 ± 1.1% for M-pH-SA, and 25.5 ± 4.1% for M-T-SA.

#### 3.2.7. Hardness Analysis

**Figure 11:**
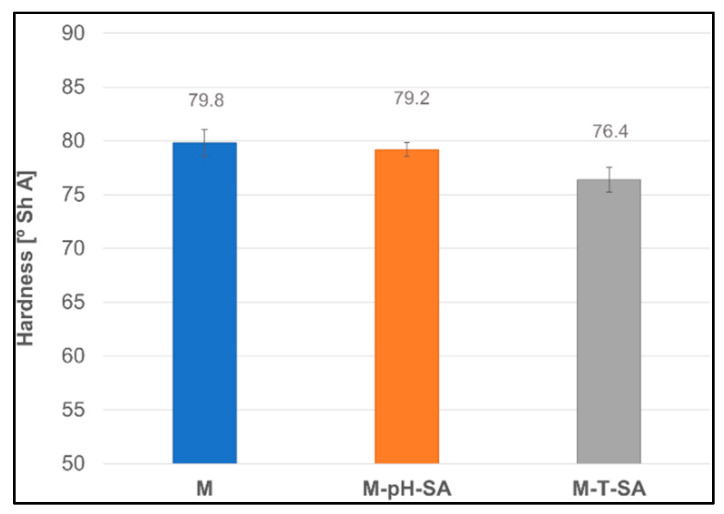
Shore’s A hardness of the bio-hybrid hydrogels (*n* = 5).

A hydrogel fabricated as a potential wound-healing dressing should be characterized by an optimum hardness so that it does not interfere with the natural healing process by tightly adhering to the wound surface. Therefore, the hardness parameter can be considered as a factor to estimate its adhesion to the skin and wound surface [67]. The hardness measurements of the fabricated bio-hybrid hydrogels are shown in Figure 11 and are approximately 80 units for the basic matrix (M). The lowest hardness value (76.4 ± 1.1) was determined for the sample, which contains thermosensitive nanocarrier–salicylic acid (M-T-SA). However, the hardness does not depend on the type of used drug carrier. These data are consistent with the obtained tensile test, which strictly affect the hardness parameter.

#### 3.2.8. The Release Profiles of Salicylic Acid from Thermosensitive Nanocarrier and Biohybrid Hydrogels

**Figure 12:**
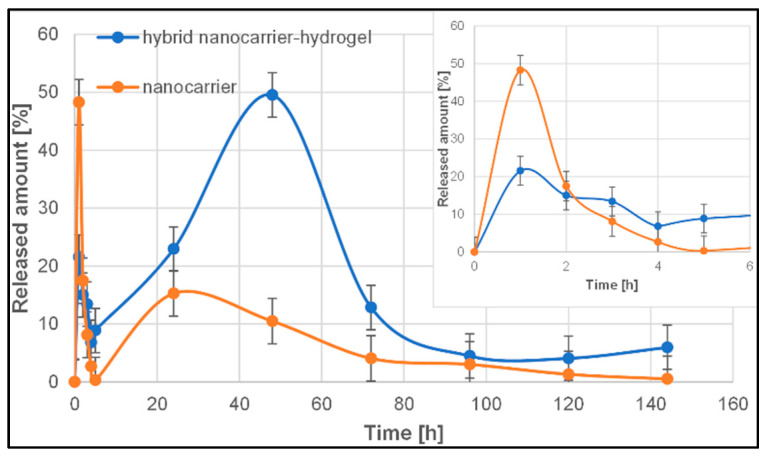
Comparison between the release profile of salicylic acid from thermosensitive nanocarrier (orange line, T-SA) and bio-hybrid hydrogel matrix (blue line, M-T-SA), at pH = 7.4 and T = 37 °C.

As the literature reports, the most common drug release profile for polymeric drug delivery systems is, in fact, a triphasic profile, and is typically observed for macromolecular drug delivery systems, or in the case of smaller particles, sometimes it can be biphasic. However, the release behaviour of polymeric drug delivery systems depends on a wide range of physicochemical parameters and should be investigated individually [68,69]. The release profiles of salicylic acid from thermosensitive nanocarrier and biohybrid hydrogels are presented in Figure 12.

There are not many reports on the release of salicylic acid from a polymeric thermosensitive nanocarrier (poly(*N*-isopropylacrylamide)). Ji et al. [70] investigated the kinetics of salicylic acid release from chitosan-based polymer carriers. The release profiles appeared to have three phases. The first phase was a rapid release or burst release in the prior period, with 45% of the salicylic acid being released in this phase (within the first 4 h). The second phase was a relatively slow release ranging from 4 to 12 h and resulted in the release of 87% of the drug. The third phase was a slower releasing process. Notably, the experiment was conducted by static method and the release time was 70 h.

In the case of our studies, the release profile of salicylic acid from a thermosensitive nanocarrier (poly(*N*-isopropylacrylamide)) (Figure 12) also consists of three phases. In the first stage, we observed the burst release effect (a significant proportion of the encapsulated drugs is rapidly released within a short period following application) without the so-called lag time. Almost 50% of the drug was released in the first 2 h in this phase. This phenomenon may be attributed to the fraction of the drug that is adsorbed or weakly bound to the large surface area of the polymer nanoparticles [71]. FT-IR analysis confirmed weak hydrogen bonds between the salicylic acid and polymeric nanoparticles (Figure S3). The second, much smaller “burst” of the drug from the carrier took place after 20 h of the experiment, while in the third phase, there was a slow-release process of the drug from the carrier, lasting up to 144 h (6 days), probably caused by the diffusion of the drug from the polymer nanoparticles through pores or erosion, which leads to pore formation and erosion effects [68,71].

The rapid burst of the drug is usually an undesirable phenomenon because it shortens the overall duration of the drug’s therapeutic effect, and excessive burst release may even cause toxicity [69]. On the other hand, the initial burst may be helpful to treat inflammation in a short time. The elimination of the rapid-release effect of the drug can be ensured by introducing it into the hydrogel matrix, which will significantly slow down this process [72].

To avoid or reduce the burst effect below the level that caused the side effects of salicylic acid, the drug-loaded polymeric nanoparticles have been incorporated into the polymeric matrix system composed of sodium alginate, poly(vinyl alcohol), and *Aloe vera*. The incorporation of the nanocarrier into the polymer matrix significantly changed the salicylic acid release profile (Figure 12). The first stage of the so-called burst release is 2-times smaller than it was with the nanocarrier itself (22% vs. 48%). It is followed by a lag time, during which the drug is gradually released over 20 h. A slow second phase or lag phase could suggest a densely packed polymer system with low porosity, which has been confirmed by SEM analysis (Figure 5). This effect could also result from pore closure and polymer-drug interactions [68]. In the next phase, there was an accelerated release phase. About 50% of salicylic acid was released in this phase after 40 h. The second burst release or a fast phase III could be attributed to erosion effects, but they could also result from polymer disintegration or cracks in the matrix [68]. The final phase is slow release controlled by slow diffusion of the drug through the polymer matrix or the existing pores and coincides with hydrolysis and degradation of the polymer [68].

It is worth emphasizing that in the case of most studies concerning the determination of release profile from polymeric vehicles, the static method was used, and the maximum analysis time was 24 h [70,72,73]. In the case of the flow method (USP 4 apparatus), the carrier with the drug was subjected to the acceptor fluid flow continuously, maintaining the so-called “sink condition”, which facilitated the continuous release of active substances from the carrier, preventing reaching an equilibrium state, thus simulating in vivo conditions. As the literature reports, this method is a great solution for determining the in vitro release of prolonged-release formulation (even up to a few days) [74,75].

#### 3.2.9. Microbiology Results

In order to investigate the influence of salicylic acid, similar tests were carried out. Table 4 presents the results of the inhibition growth of the chosen microorganisms caused by the used materials.

**Figure 13:**
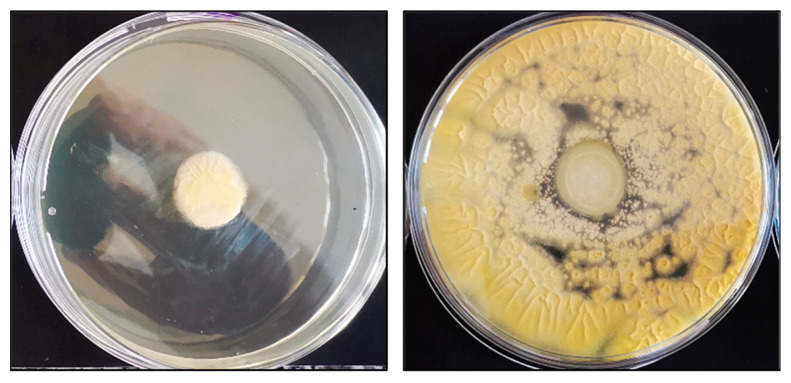
Photographs after microbiology tests.

**Figure 14:**
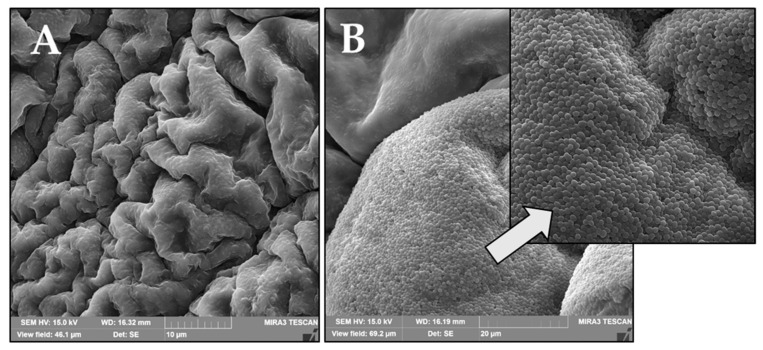
SEM microphotography of bio-hybrid hydrogels: M-T-SA (**A**) and basic matrix (M) (**B**).

From the conducted microbiological tests using four strains of cells exposed to the prepared compositions, it can be seen that the selected samples showed antibacterial activity, while in relation to yeast and fungi cells, the results showed no visible influence on the growth of the cells. It can be observed that all of the used materials with the inserted drug showed antibacterial activity again both *S. aureus* and *E. coli* (Figure 13). Those two strains are the main pathogens associated with acute wounds [76]. Moreover, the selected samples, such as base matrix (M) and M-T-SA, after microbiological tests on *S. aureus* bacteria, were analyzed using SEM (Figure 14).

As can be seen on the right side in Figure 14B of the SEM image, the surface of the base matrix (M) is completely covered with yeast cells, which shows that without the addition of the drug, it has no antimicrobial activity. Additionally, it can be noticed that the *S. aureus* bacteria appear as spherical and form in grape-like clusters [77]. However, the surface of bio-hybrid hydrogel containing T-SA is clear without any additional bacterial colonies. It confirmed that salicylic acid was released, and it inhibited culture growth.

## 4. Conclusions

To sum up, bio-hybrid hydrogels containing the thermo/pH-sensitive nanocarriers–salicylic acid system (T-SA/pH-SA) were prepared successfully, which was confirmed based on different test results. The average particle size of empty pH- and thermosensitive carriers is about 479 nm and 118 nm, respectively, while after the encapsulation of salicylic acid, these values increase up to 700 and 356 nm for pH-SA and T-SA, respectively. The obtained results indicate that incorporating the system of T-SA or pH-SA into the hydrogel matrix does not significantly influence the physicochemical properties of the final materials, such as swelling ability and degradation studies. Moreover, TG and DTG curves of the bio-hybrid hydrogels show that salicylic acid-modified nanocarriers increased the thermal stability of the systems compared to the unmodified basic matrix. Based on the DSC curves, no exothermic peaks were observed, which means that no post-crosslinking processes are taking place, and the bio-hybrid hydrogels are well cross-linked. FTIR analysis allows concluding that introducing the thermo/pH-sensitive nanocarriers–salicylic acid system into the basic matrix does not change the chemical structure of the hydrogels. Results show also that incorporating a carrier–drug system into the matrix does not deteriorate the mechanical properties. The values of the maximum elongation at break of the unmodified hydrogel (M) was ~27%, while it was ~25% for the pH-sensitive nanocarrier–salicylic acid hydrogel and 26% for the thermosensitive nanocarrier–salicylic acid hydrogel.

The incorporation of the system of T-SA into the matrix significantly changed the course of the salicylic acid release profile, which was determined using the USP4 method. The first stage of the so-called burst release is 2-times smaller than it was with the nanocarrier itself (22% vs. 48%). About 50% of salicylic acid was released in this phase after 40 h.

All of the fabricated materials containing the thermo/pH-sensitive nanocarrier system with salicylic acid (T-SA/pH-SA) showed antibacterial activity against both *S. aureus* and *E. coli*.

The presented developed method for obtaining bio-hybrid hydrogels with a drug–nanocarrier system has a real chance of becoming a commercial method of treating skin wounds. However, it is not possible without further in vitro and in vivo tests and the selection of an appropriate sterilization method for the final materials. For today, a limitation may also be the increase in the scale of the potential production of bio-hybrids. Despite the above limitations, we are currently working on developing bio-hybrid materials containing a variety of active substances/drugs, dual drug systems, and their expected release profiles in a living body.

## Figures and Tables

**Table 1:** The average particle size (nm) of the obtained empty thermo/pH-sensitive carriers and their systems with SA.

Sample	Average Particle Size (nm)
T	118
T-SA	356
pH	479
pH-SA	709

**Table 2:** Encapsulation efficiency (%) of the obtained systems of nanocarrier–salicylic acid.

Sample	Encapsulation Efficiency (%)
T-SA	80.64
pH-SA	78.48

**Table 3:** Thermal properties of bio-hybrid hydrogels and substrates used for their production.

Sample Symbols	T_5_	T_10_	T_50_	Residual Mass at 800 °C
	(°C)			(%)
T	53.3	143.5	318.6	11.66
pH	76.8	213.4	367.5	12.56
SA	141.6	151.5	177.5	0.00
T-SA	119.9	131.3	166.3	4.10
pH-SA	150.0	183.8	387.4	7.67
M	134.1	179.5	378.8	9.39
M-T-SA	146.5	186.8	386.9	10.04
M-pH-SA	144.3	183.3	388.1	9.2

**Table 4:** The inhibition growth of the chosen microorganisms caused by the used materials.

Sample	*Staphylococcus aureus*	*Aspergillus niger*	*Candida albicans*	*Esherichia coli*
T	+	−	−	+
pH	+	−	−	+
SA	+	−	−	+
T-SA	+	−	−	+
pH-SA	+	−	−	+
M	−	−	−	−
M-T-SA	+	−	−	+
M-pH-SA	+	−	−	+

+ inhibition present; − lack of inhibition.

## Data Availability

The data that support the findings of this study are contained within the article.

## Supplementary Materials

The following are available online at https://www.mdpi.com/article/10.3390/pharmaceutics14040773/s1, Figure S1: The average particle size distribution of the system of pH-sensitive nanocarriers-salicylic acid. Figure S2: The average particle size distribution of the system of thermosensitive nanocarriers-salicylic acid. Figure S3: FT-IR spectra of the system of T-SA and pH-SA, salicylic acid (SA), and *Aloe vera* (AV).
