# Supplementary material for: Bio-Hybrid Hydrogels Incorporated into a System of Salicylic Acid-pH/Thermosensitive Nanocarriers Intended for Cutaneous Wound-Healing Processes
Source: Pharmaceutics. 2022 Apr 1;14(4):773. doi: 10.3390/pharmaceutics14040773 (PMC9031596; doi:10.3390/pharmaceutics14040773)
Supplement: Supplementary file 1 [file pharmaceutics-14-00773-s001.zip › pharmaceutics-1646024-supplementary.pdf]

pharmaceutics

MDPI

Supplementary Materials: Bio-Hybrid Hydrogels Incorporated into a System of Salicylic Acid– pH/Thermosensitive Nanocarriers Intended for Cutaneous Wound-Healing Processes

Katarzyna Bialik-Wąs, Małgorzata Miastkowska, Paulina Sapuła, Klaudia Pluta, Dagmara Malina, Jarosław Chwastowski and Mateusz Barczewski

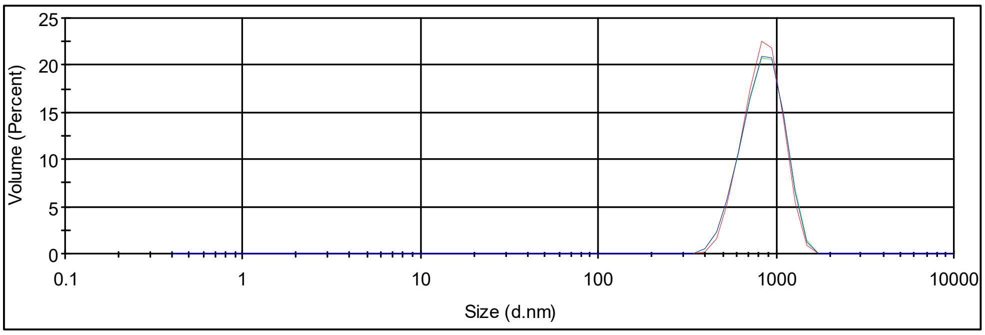

Figure S1. The average particle size distribution of the system of pH-sensitive nanocarriers-salicylic acid.

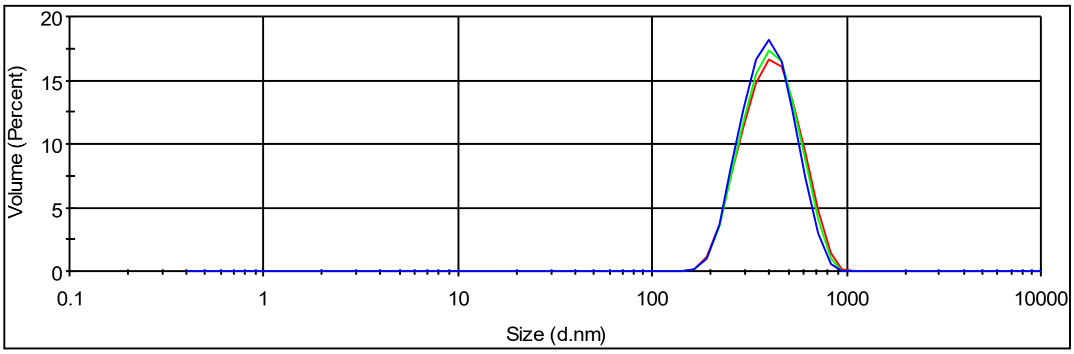

Figure S2. The average particle size distribution of the system of thermosensitive nanocarriers-salicylic acid.

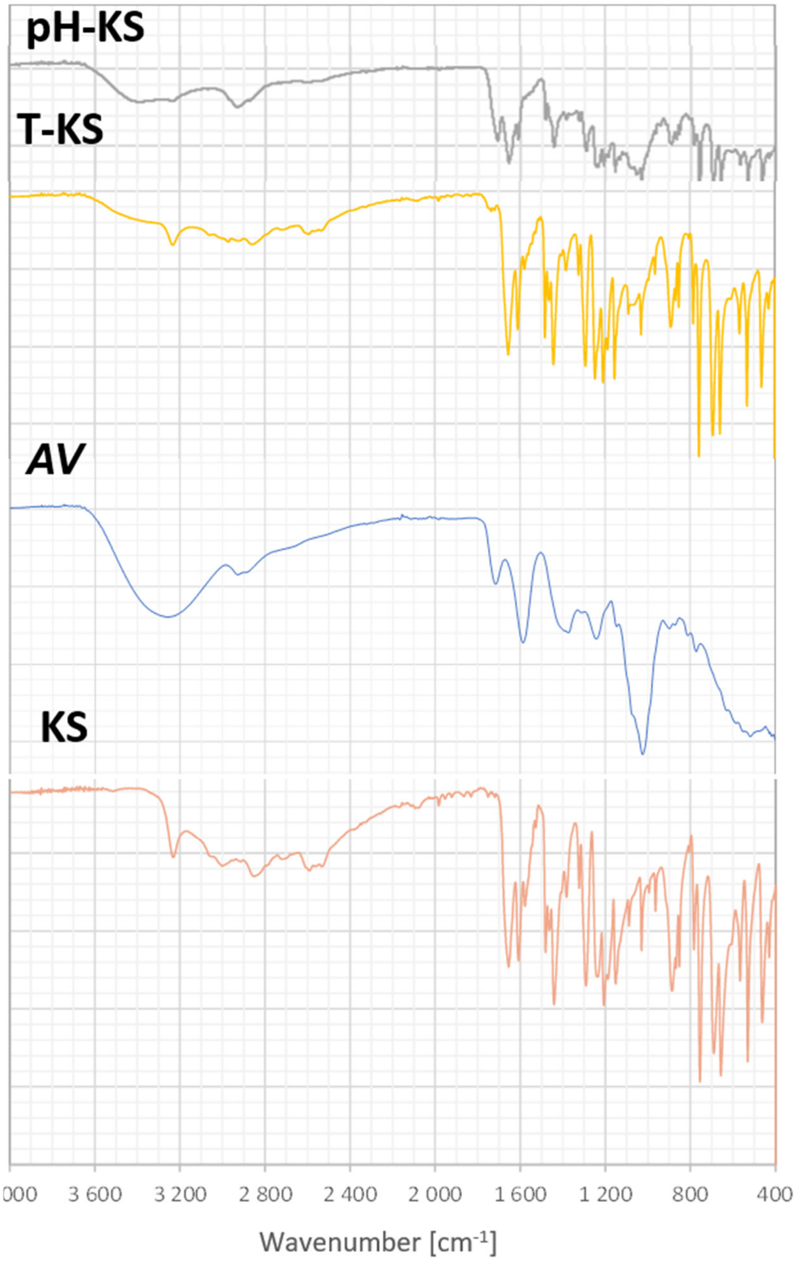

Figure S3. FT-IR spectra of the system of T-SA and pH-SA, salicylic acid (SA), and *Aloe vera* (*AV*).
